# Immune checkpoint inhibition in patients treated with stereotactic radiation for brain metastases

**DOI:** 10.1186/s13014-020-01644-x

**Published:** 2020-10-27

**Authors:** Emily S. Kowalski, Jill S. Remick, Kai Sun, Gregory S. Alexander, Rahul Khairnar, Emily Morse, Hua-Ren Cherng, Lars J. Berg, Yannick Poirier, Narottam Lamichhane, Stewart Becker, Shifeng Chen, Jason K. Molitoris, Young Kwok, William F. Regine, Mark V. Mishra

**Affiliations:** 1 Department of Radiation Oncology, University of Maryland School of Medicine, 850 W. Baltimore Street, Baltimore, MD 21202 USA; 2 Department of Radiation Oncology, University of Maryland Medical Center, Baltimore, MD USA; 3 Department of Pharmaceuticals Health Services Research, University of Maryland School of Pharmacy, Baltimore, MD USA; 4 University of Maryland School of Medicine, Baltimore, MD USA

## Abstract

**Purpose:**

Stereotactic radiation therapy (SRT) and immune checkpoint inhibitors (ICI) may act synergistically to improve treatment outcomes but may also increase the risk of symptomatic radiation necrosis (RN). The objective of this study was to compare outcomes for patients undergoing SRT with and without concurrent ICI.

**Methods and materials:**

Patients treated for BMs with single or multi-fraction SRT were retrospectively reviewed. Concurrent ICI with SRT (SRT-ICI) was defined as administration within 3 months of SRT. Local control (LC), radiation necrosis (RN) risk and distant brain failure (DBF) were estimated by the Kaplan-Meier method and compared between groups using the log-rank test. Wilcoxon rank sum and Chi-square tests were used to compare covariates. Multivariate cox regression analysis (MVA) was performed.

**Results:**

One hundred seventy-nine patients treated with SRT for 385 brain lesions were included; 36 patients with 99 lesions received SRT-ICI. Median follow up was 10.3 months (SRT alone) and 7.7 months (SRT- ICI) (*p* = 0.08). Lesions treated with SRT-ICI were more commonly squamous histology (17% vs 8%) melanoma (20% vs 2%) or renal cell carcinoma (8% vs 6%), (*p* < 0.001). Non-small cell lung cancer (NSCLC) compromised 60% of patients receiving ICI (*n* = 59). Lesions treated with SRT-ICI had significantly improved 1-year local control compared to SRT alone (98 and 89.5%, respectively (*p* = 0.0078). On subset analysis of NSCLC patients alone, ICI was also associated with improved 1 year local control (100% vs. 90.1%) (*p* = 0.018). On MVA, only tumor size ≤2 cm was significantly associated with LC (HR 0.38, *p* = 0.02), whereas the HR for concurrent ICI with SRS was 0.26 (*p* = 0.08). One year DBF (41% vs. 53%; *p* = 0.21), OS (58% vs. 56%; *p* = 0.79) and RN incidence (7% vs. 4%; *p* = 0.25) were similar for SRT alone versus SRT-ICI, for the population as a whole and those patients with NSCLC.

**Conclusion:**

These results suggest SRT-ICI may improve local control of brain metastases and is not associated with an increased risk of symptomatic radiation necrosis in a cohort of predominantly NSCLC patients. Larger, prospective studies are necessary to validate these findings and better elucidate the impact of SRT-ICI on other disease outcomes.

## Introduction

Multiple studies have established stereotactic radiation therapy (SRT) as the preferred non-surgical treatment for patients with limited brain metastases (BMs) given the increased risk of neurocognitive decline following whole brain radiotherapy (WBRT) [1–7]. More recent literature suggests improved survival with SRT over WBRT in the context of re-treatment upon intracranial progression [8]. As the number of patients with metastatic cancer receiving immune checkpoint inhibitors (ICIs) continues to increase, the interactions between ICIs and SRT require investigation.

Several prospective trials have demonstrated activity of ICIs in the brain, with response rates of ranging from 26% to 57% in absence of brain radiotherapy [9, 10]. SRS/SRT has been found to act synergistically with ICIs through multiple mechanisms including stimulating the release of tumor antigens, enhancing activation of antigen presenting cells, increasing the permeability of the blood-brain barrier and upregulating cell-surface molecules targeted by ICIs [11, 12]. Several retrospective studies, primarily involving patients with melanoma, have demonstrated an improvement in disease outcomes associated with SRT and concurrent ICI administration [13–15]. However, there remains concern that concurrent SRT and ICI may also lead to an increased risk of symptomatic toxicity given that normal tissues also express PD-L1 to prevent T-cell mediated damage of normal tissue [16].

A retrospective study evaluated 80 patients with melanoma who received ICI within one week of SRS for brain metastases suggested that SRS with concurrent ICI may improve treatment outcomes, however, potentially at the expense of an increased risk of developing symptomatic radiation necrosis (RN) [17]. Other single institutional series have corroborated these findings. For example, Helis and colleagues reported an increased risk of adverse radiation events including subacute edema, pseudoprogression and radiation necrosis at 2 years (4.5% vs 2.1%) in their population of patients treated with frame-based SRS with and without concurrent ICI, respectively [18]. In a population of patients. with non-small cell lung cancer (NSCLC), melanoma and renal cell carcinoma, Martin et al. also found that patients treated with ICIs and SRS/SRT were significantly more likely to experience symptomatic radiation necrosis and this association was most predominant in patients with melanoma [19].

The purpose of this study is to compare outcome for patients with BMs treated with single-fraction stereotactic radiosurgery (SRS) or multiple-fraction (MF) SRT with or without concurrent ICIs within a single academic network of radiation oncology centers.

## Materials and methods

A retrospective multi-center review was conducted of patients treated for BMs with SRS or MF-SRT within an academic network. Concurrent ICI was defined as administration of ICI in the 3 month period leading up to or immediately after SRS or SRT. ICIs included: Anti cytotoxic T-lymphocyte associated protein 4 (CTLA-4, Ipilimumab), anti-programmed cell death receptor (PD-1, Pembrolizumab, Nivolumab) and anti-programmed cell death ligand (PD-L1, Durvalumab, Atezolizumab). Eligible patients had undergone diagnostic MRI and at least one post-SRT MRI. Endpoints analyzed included local control, distant brain control, overall survival and radiation necrosis.

### Treatment planning and delivery

SRS was performed with either frame-based cobalt radiosurgery delivery device or a frameless linear accelerator (LINAC) based technique with a robotic 6-degree of freedom couch. MF-SRT was delivered using LINACs.

Gross tumor volume was defined as the contrast enhancing tumor volume on T1 axial MRI fused with a treatment planning CT scan. For LINAC-based treatments, a clinical tumor volume margin was added when treating the cavity in cases of prior resections. A planning target volume (PTV) margin was added at physician discretion based on setup uncertainty. There was no PTV margin used for lesions treated with frame-based SRS. For SRS, prescription dose was dependent on the lesion size as follows: ≤ 2 cm (21–24 Gy), 2-3 cm (18 Gy) and 3–4 cm (15 Gy). Dosing for post-operative SRS was determined by surgical cavity volume. For MF-SRT, dosing regimens were at the discretion of the treating physician. The prescription dose was prescribed to the 50% isodose line for frame-based SRS and the 80% isodose line for those lesions treated with the LINAC.

### Patient follow-up

Clinical assessment and MRI surveillance occurred 1 month after treatment and every 3 months thereafter. Local failure (LF) and radiation necrosis (RN) were documented based on clinical assessment, imaging findings and pathology. Tumor progression required 2 consecutive MRIs with increased T1 contrast enhancement and increased vascular flow on perfusion MRI, when available. Criteria for radiation necrosis included: 1) increased T1 enhancement in the high dose radiation field associated with increased peripheral edema and a central region of hypo-intensity, 2) a decrease or resolution of enhancement on subsequent follow up imaging, and 3) absence of increased vascular flow on perfusion-weighted MRI sequences.

### Statistical analysis

The primary endpoints of this study were LF and incidence of RN. Time to LF and RN were defined from treatment start date to event or last follow-up date. LC, DBF and RN probability were estimated by the Kaplan-Meier method and compared between groups using the log-rank test. Patients were censored at date of last follow-up or death, or at date of salvage WBRT (for LC and RN endpoints). Characteristics of patients treated with and without concurrent ICI were compared using Wilcoxon rank sum tests for continuous and chi-square tests for categorical variables. Covariates that were clinically relevant to the outcome of interest or significant on univariate analysis were included in the multivariate model. Subset analyses of non-small cell lung cancer (NSCLC) lesions and timing of immunotherapy delivery were performed. SAS (version 9.4, SAS Institute, Cary, NC) statistics software was utilized. All statistical analyses were performed at a significance level of 0.05.

## Results

### Patient and tumor characteristics

Three hundred eighty-five treated brain lesions from 179 patients were included. Ninety-nine lesions were treated with SRS or SRT with ICI and 286 lesions received SRS or SRT alone. Patient and lesion characteristics are shown in Table 1. ICIs administered included Pembrolizumab (*n* = 44, 44%), Nivolumab (*n* = 34, 34%), Ipilimumab (*n* = 8, 8%), Atezolizumab (*n* = 5, 5%), Durvalumab (*n* = 3, 3%), both Nivolumab and Ipilimumab (*n* = 4, 4%), unknown (*n* = 1, 1%). Compared to patients treated with radiation alone, patients treated with radiation and ICI more commonly had squamous histology (17% vs 8%), melanoma (20% vs 2%) or renal cell carcinoma (8% vs 6%) (*p* < 0.001). In addition, lesions treated with SRS + ICI were less frequently treated with cytotoxic systemic therapy (11% vs 24%; *p* = 0.007) or prior WBRT (22% vs 38%)(*p* = 0.04).

**Table 1:** Patient and Lesion Characteristics

**Variables by patient**	**SRS alone (%)** ***N*** **= 143**	**SRS-ICI (%)** ***N*** **= 36**	***P*-value**
**Median Follow up (q1, q3)**	10.3 (5.8, 12.0)	7.7 (4.2, 12.0)	**0.08**
**Median age (q1, q3)**	60 (54, 67)	59 (55, 71)	**0.65**
**Race**			
White	95 (66)	26 (72)	0.51
Other	48 (34)	10 (28)	
**KPS**			
80–100	105 (73)	29 (81)	0.38
50–70	38 (27)	7 (19)	
**Primary Site**			
Lung	75 (52)	22 (61)	0.35
Other	68 (48)	14 (39)	
**Histology**			
Renal cell	9 (6.3)	3(8.3)	**< 0.001**
Melanoma	3 (2.1)	7 (19.5)	
Squamous cell	11(7.7)	6 (16.7)	
Adenocarcinoma	91 (63.6)	17 (47.2)	
Other	29 (20.3)	3 (8.3)	
**Active Extra-CNS Disease**			0.36
Extensive	62 (43)	20 (56)	
Bone only	8 (6)	3 (8)	
Primary only	41 (29)	9 (25)	
None	32 (22)	4 (11)	
**Prior WBRT**			0.01
Yes	49 (34)	4 (11)	
No	94 (66)	32 (89)	
**Variables by lesion**	**SRS alone (%)** ***N*** **= 286**	**SRS-ICI (%)** ***N*** **= 99**	***P*-value**
**Tumor size**			0.03
≤ 2 cm	226 (79)	88 (89)	
> 2 cm	60 (21)	11 (11)	
**SRS fractions**			
Single fraction	203 (71)	77 (78)	0.19
Multi-fraction	83 (29)	22 (22)	
**Surgical Resection**			
No	265 (93)	93 (94)	0.67
Yes	21 (7)	6 (6)	
**Concurrent Systemic therapy**			**0.007**
Yes	68 (24)	11 (11)	
No	218 (76)	88 (89)	
**Prior WBRT**			**0.004**
Yes	108 (37.8)	22 (22.2)	
No	178 (62.2)	77 (77.8)	
**Histology**			< 0.001
Renal cell	14 (4.9)	15 (15.1)	
Melanoma	12 (4.2)	20 (20.2)	
Squamous cell	18 (6.3)	10 (10.1)	
Adenocarcinoma	185 (64.7)	48 (48.5)	
Other	57 (19.9)	6 (6.1)	
**Median Prescription Dose for SRS (single fraction)**	21 (20, 24)	24 (21, 24)	< 0.001
**Median Biologically Equivalent Dose for SRT (multiple fraction)**	38 (36, 43)	50 (38, 51)	0.002

### Treatment outcomes and toxicity

**Fig. 1:**
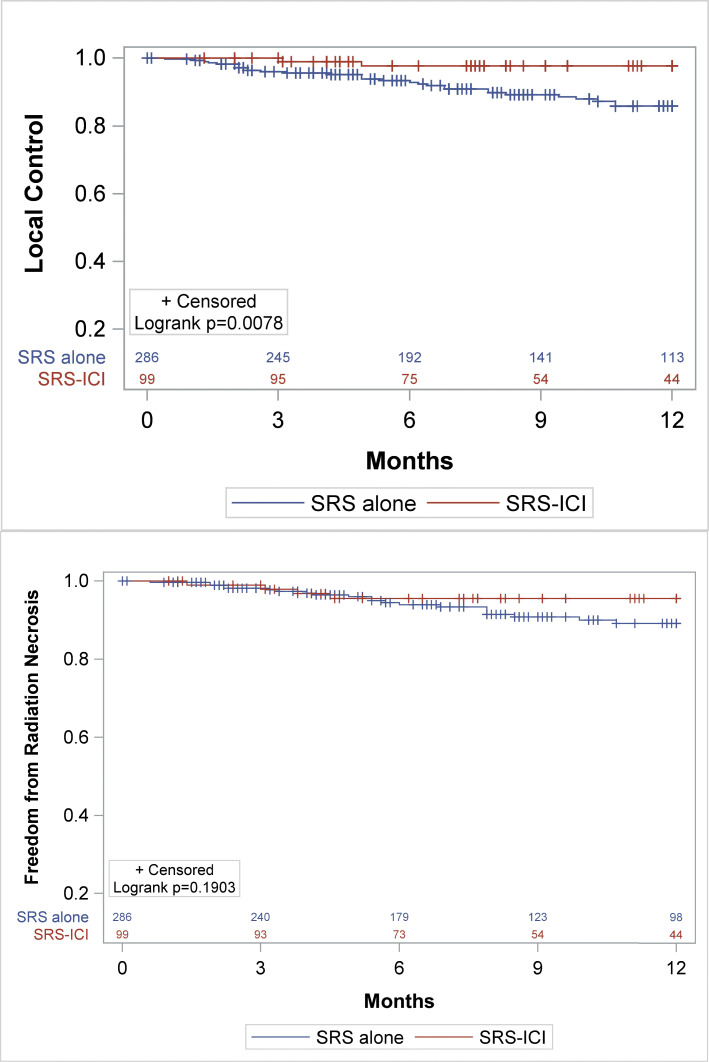
Kaplan Meier comparison between SRS or SRT alone (blue) and SRS/SRT-ICI (red) in terms of **a** Local control, **b** Radiation necrosis-free survival

**Fig. 2:**
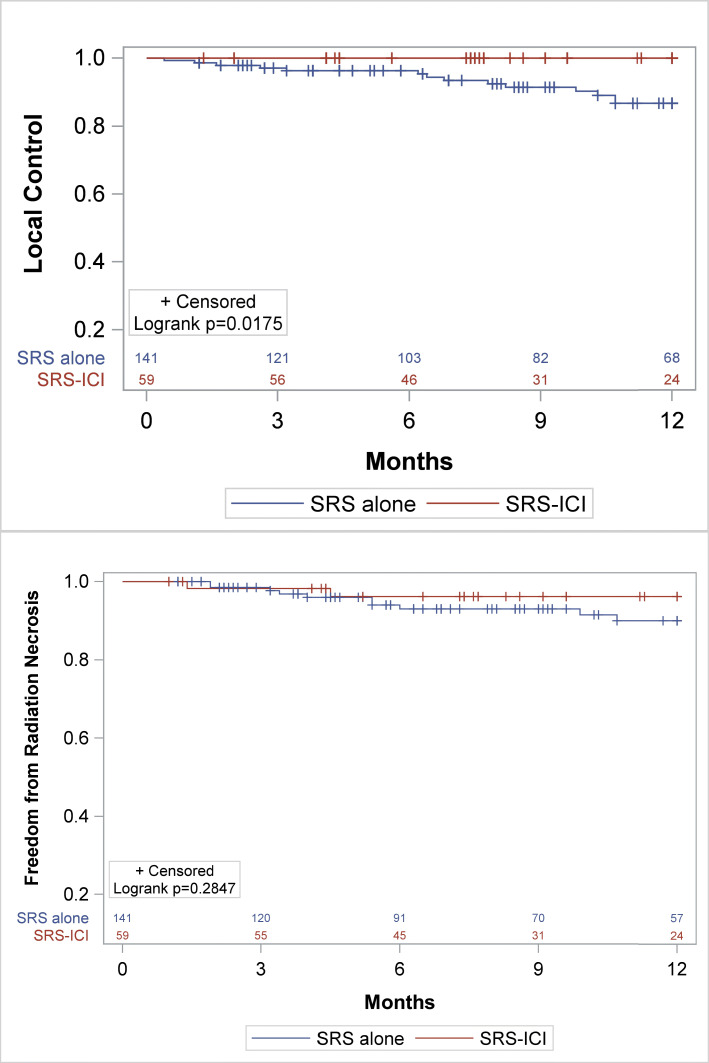
Subset analysis of NSCLC patients. Kaplan Meier comparison between SRS or SRT alone (blue) and SRS/SRT-ICI (red) in terms of **a** Local control, **b** Radiation necrosis-free survival

The median follow-up time was 10.3 months (SRS/SRT alone) and 7.7 months (SRS/SRT-ICI), (*p* = 0.08). Patients who received SRS/SRT-ICI had significantly improved Kaplan-Meier estimates for local control (98.0% vs. 89.5%, *p* = 0.0078, Fig. 1a). A similar 1-year local control benefit (100% vs. 90.1%) was also observed in the subset of NSCLC lesions (*n* = 59, 60%) treated with SRS or SRT and ICI (Fig. 2a, *p* = .0175). On UVA, ICI (HR 0.18, *p* = 0.02) and tumor size ≤2 cm (HR 0.36, *p* = 0.01) significantly reduced the risk of local failure (Table 2). On MVA, tumor size was significantly correlated with reduced LF (HR 0.38, *p* = 0.02), whereas SRT-ICI was not (HR 0.26, *p* = 0.08**).** One-year DBF (53% SRS/SRT-ICI vs 41% SRS/SRT alone; *p* = 0.21) and OS (56% SRS/SRT-ICI vs 58% SRS/SRT alone; *p* = 0.79) did not differ between those treated with or without ICI. Subset analysis of the patients with NSCLC confirmed no significant difference in 1-year DBF (59% SRS/SRT-ICI vs. 44% SRS/SRT alone, *p* = 0.21) or 1-year OS (64% SRS/SRT-ICI vs 65% SRS/SRT alone, *p* = 0.88) between the two groups.

**Table 2:** Univariate and multivariate analysis for local failure

**Covariate**	**HR (95% CI)**	***P*-value**
*Univariate*		
**Surgical resection**	1.44 (0.44–4.75)	0.55
**Tumor size ≤2 cm**	0.36 (0.17–0.75)	0.01
**Concurrent systemic therapy**	1.37 (0.64–2.97)	0.42
**Location of metastasis**	1.10 (0.47–2.54)	0.83
**Histology**		
Renal cell	0.40 (0.04–3.55)	0.41
Melanoma	0.34 (0.04–3.09)	0.22
Squamous cell	1.67 (0.37–7.48)	0.50
Adenocarcinoma	1.23 (0.42–3.56)	0.71
**Recent Immunotherapy**	0.18 (0.04–0.75)	0.02
**Prior WBRT**	1.88 (0.94–3.76)	0.08
*Multivariate*		
**Recent immunotherapy**	0.26 (0.06–1.17)	0.08
**Prior WBRT**	1.66 (0.75–3.67)	0.21
**Tumor size ≤2 cm**	0.38 (0.17–0.84)	0.02

**Fig. 3:**
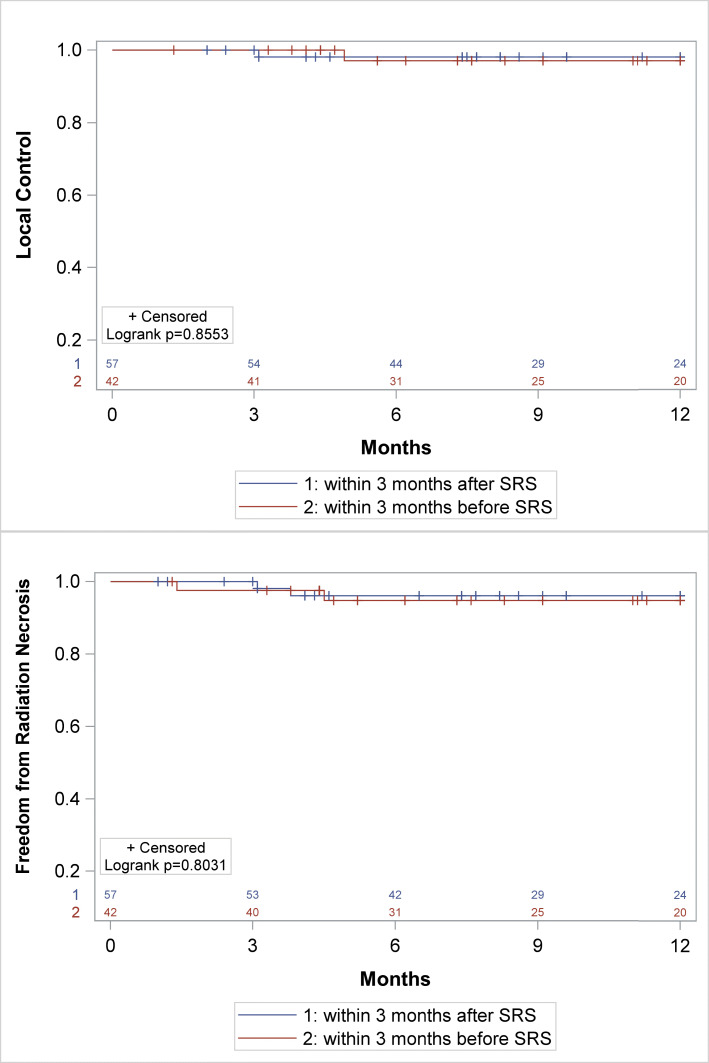
Subset analysis of timing of administration of ICI. Kaplan Meier comparison between administration of ICI within 3 months after SRS/SRT (blue) or administration of ICI within 3 months before SRS/SRT (red) in terms of **a** Local control, **b** Radiation necrosis-free survival

The 1-year incidence of symptomatic RN were similar for SRS/SRT-ICI and SRS/SRT alone at 4.0% and 7.3%, respectively (*p* = 0.25) (Fig. 1b), and was also similar on a subset analysis of only NSCLC lesions (3.4% SRS/SRT -ICI vs. 7.1% SRS/SRT alone, *p* = 0.31, Fig. 2b). Only tumor size ≤2 cm predicted for RN on both UVA and MVA analysis (Table 3). Initiation of ICI in relation to RT (initiated within 3 months before versus within 3 months after SRT) did not impact local control (Fig. 3a) or radiation necrosis (Fig. 3b).

**Table 3:** Univariate and multivariate analysis for radiation necrosis

**Covariate**	**HR (95% CI)**	***P*-value**
*Univariate*		
Surgical resection	1.92 (0.57–6.46)	0.29
Tumor size ≤2 cm	0.28 (0.13–0.63)	0.002
Concurrent systemic therapy	1.03 (0.41–2.59)	0.94
Location of metastasis	1.23 (0.46–3.29)	0.68
Single vs Multi-fraction SRT	1.24 (0.52–2.96)	0.64
Age	0.99 (0.95–1.02)	0.47
Recent Immunotherapy	0.50 (0.17–1.45)	0.20
Prior WBRT	1.89 (0.86–4.15)	0.11
*Multivariate*		
Recent immunotherapy	0.98 (0.28–3.51)	0.98
Prior WBRT	1.55 (0.56–4.31)	0.40
Tumor size ≤2 cm	0.24 (0.09–0.64)	0.004

## Discussion

In this multi-center experience of patients treated with SRS or SRT for brain metastases, we found no significant difference in the incidence of symptomatic RN between patients receiving SRS/SRT-ICI and those receiving SRS/SRT alone. Patients receiving SRS/SRT-ICI had a significantly improved 1-year local control rate compared to those receiving SRS or SRT alone, although there was only a trend towards significance on MVA. In contrast to previous studies, we found no differences in the rate of DBF or OS between the two cohorts [13, 14].

Unique to our study, the majority of lesions treated with anti-PDL1 therapies were NSCLC, whereas most series of concurrent ICI and SRS/SRT include predominantly melanomatous brain lesions. A subset analysis also demonstrated improved 1-yr local control when NSCLC lesions received SRS or SRT with ICI**.** It has been shown that patients with NSCLC treated with ICI and brain-directed therapy have inferior survival compared to other histologies [20]. Patients with NSCLC treated in this series may have succumbed to their systemic disease or competing risk factors for death before experiencing a distant brain failure. This is one factor which may explain the lack of benefit in distant brain control and overall survival with SRS/SRT -ICI in our study as compared to others. Overall, it appears that patients treated with SRS or SRT and ICI were treated more recently (range: 2014–2019) than patients treated with SRS or SRT alone (range: 2008–2019). This time discrepancy correlates with the recent surge in usage of ICI to treat metastatic malignancies [13, 15]. MRI technology available to patients at our centers has also improved dramatically over a similar time period. The cohort receiving SRS or SRT with ICI likely received more modern surveillance MRIs after SRS or SRT with thinner image slices and volumetric imaging. Therefore distant brain failures were likely to be more readily detected in this more modern cohort receiving ICI.

A local control benefit with ICI has been less commonly observed. Analogous to our study, Chen et al. reported a significant improvement in local progression-free survival with concurrent anti-PD1 therapy which lost its significance on MVA [15]. Similarly, Cohen-Inbar et al. reported a significant improvement in local control among patients treated with SRS before or during anti-CTLA4 therapy cycles compared to patients that received SRS after ICI [14].

The definition of concurrent ICI in the literature has been inconsistent making it difficult to draw broad conclusions regarding the optimal timing and sequencing of SRS/SRT and ICI. Chen et al. demonstrated a significant survival benefit among patients receiving ICI within a 2 week window of SRS/SRT [15]. A more recent study reported a significant improvement in overall brain response and durability of response when SRS was delivered within 5 half-lives of the ICI, with the greatest response observed within 1 month [21]. Qian and co-investigators also demonstrated greater percentage reduction in volume of melanoma brain lesions when ICI was given within 1 month of SRS [22]. A recent meta-analysis of 17 studies revealed local control and survival was significantly improved when ICI was given concurrently versus non concurrently; however the definition of “concurrent” was not standardized from study to study [23]. The sequencing of SRS/SRT and ICI appears to be important with several studies demonstrating a greater clinical benefit when radiation is given before ICI as compared to after [13, 14]. Furthermore recent studies indicate that the optimal timing of ICI delivery may vary based on the actual agent prescribed [24]. An ongoing prospective phase II study evaluating the timing of Ipilimumab and SRS in patients with melanomatous brain lesions will hopefully shed more light on this issue (NCT02097732). Given the lack of consensus definition of concurrent immunotherapy in the present literature, in this series concurrent ICI was defined as that delivered within 3 months of SRS/SRT to encompass any potential interaction between these two therapies. No difference in local control or radiation necrosis was found when a subset analysis was performed of patients receiving ICI 3 months before or after SRS/SRT. It possible that the broad definition of “concurrent” in this study may have precluded the ability to detect a difference based on timing of administration of ICI.

Importantly, in this series concurrent ICI with SRS/SRT was not associated with an increased risk of symptomatic radiation necrosis, with a 4% rate of symptomatic RN at 1 year. This is consistent with prior reports demonstrating no increased risk of developing a high-grade neurologic toxicity or pathologically-confirmed RN with SRT-ICI [15, 23, 25]. In this study, we collected and reported rates of symptomatic RN only as this represents the most clinically relevant toxicity following radiosurgery. This experience adds to the growing body of evidence suggesting that combining these two modalities is not associated with increased risks for toxicity.

There are several limitations of this retrospective analysis. Patients included may have received prior systemic therapies and corticosteroids at different durations and doses which may have impacted the efficacy of immunotherapy. In this study, concurrent ICI was defined as within a 3 month window either before or after SRT. Given the half-life of Anti-PDL-1 agents is approximately 25 days, the time period utilized in this study may have mitigated the potential distant control benefit and overall survival benefit of concurrent ICI. In this study, cases of pseuodoprogression were not distinguished from radiation necrosis primarily due to the significant challenge in distinguishing between these two diagnoses with imaging alone. Without pathologic confirmation, the main differentiator between these two diagnoses is time, with pseudoprogression most often occurring in the first 12 weeks whereas radiation necrosis most often occurs 3 months to several years after SRT [26]. Similar to grade 1 radiation necrosis, Pseudoprogression is also generally asymptomatic [26]. Therefore, to minimize the impact of these confounding diagnoses on our analysis as much as possible, we chose to limit our reporting of RN to those that were symptomatic (grade 2 or higher). This study did not include pathologic evaluation of specimens and clearly there are inherent limitations when discerning between RN, pseudoprogression and local failure based on patients’ clinical presentations and imaging findings.

## Conclusion

This experience suggests that SRS/SRT -ICI is safe and may provide enhanced local control of brain metastases in a cohort of predominantly NSCLC patients. Large prospective studies are necessary to elucidate the impact of combined therapy with SRS/SRT and ICI on distant brain control and overall survival in this population as well as improve the understanding of how timing, fractionation and histology may impact the outcome of these results.

## Data Availability

All data generated or analyzed during this study are included in this published article.

## Abbreviations

SRS: Stereotactic radiosurgery
SRT: Stereotactic radiation therapy
MF-SRT: Multi-fraction stereotactic radiation therapy
ICI: Immune checkpoint inhibitors
RN: Radiation Necrosis
LC: Local control
DBF: Distant brain failure
MVA: Multivariable analysis
OS: Overall survival
NSCLC: Non-small cell lung cancer
PD-L1: Anti-programmed cell death ligand
CTLA-4: Anti cytotoxic T-lymphocyte associated protein 4
BM: Brain Metastases
SF: Single fraction
MF: Multi-fraction
WBRT: Whole brain radiation therapy
PTV: Planning target volume
UVA: Univariate analysis
HR: Hazard ratio

## References

[1] Aoyama H, Shirato H, Tago M, Nakagawa K, Toyoda T, Hatano K, et al. Stereotactic radiosurgery plus whole-brain radiation therapy vs stereotactic radiosurgery alone for treatment of brain metastases: a randomized controlled trial. J Am Med Assoc. 2006;295(21):2483–91. 10.1001/jama.295.21.2483 16757720

[2] Kocher M, Soffietti R, Abacioglu U, Villà S, Fauchon F, Baumert BG, et al. Adjuvant whole-brain radiotherapy versus observation after radiosurgery or surgical resection of one to three cerebral metastases: results of the EORTC 22952-26001 study. J Clin Oncol. 2011;29(2):134–41. 10.1200/JCO.2010.30.1655 PMC3058272 21041710

[3] Brown PD, Jaeckle K, Ballman KV, et al. Effect of radiosurgery alone vs radiosurgery with whole brain radiation therapy on cognitive function in patients with 1 to 3 brain metastases: a randomized clinical trial. JAMA. 2016;316(4):401–9. 10.1001/jama.2016.9839 PMC5313044 27458945

[4] Mahajan A, Ahmed S, McAleer MF, et al. Post-operative stereotactic radiosurgery versus observation for completely resected brain metastases: a single-centre, randomised, controlled, phase 3 trial. Lancet Oncol. 2017;18(8):1040–8. 10.1016/S1470-2045(17)30414-X PMC5560102 28687375

[5] Brown PD, Ballman KV, Cerhan JH, Anderson SK, Carrero XW, Whitton AC (2017). Postoperative stereotactic radiosurgery compared with whole brain radiotherapy for resected metastatic brain disease (NCCTG N107C/CEC·3): a multicentre, randomised, controlled, phase 3 trial. Lancet Oncol [internet]. Elsevier Ltd.

[6] Kayama T, Sato S, Sakurada K, Mizusawa J, Nishikawa R, Narita Y (2018). Effects of surgery with salvage stereotactic radiosurgery versus surgery with whole-brain radiation therapy in patients with one to four brain metastases (JCOG0504): a phase III, noninferiority, randomized controlled trial. J Clin Oncol.

[7] Yamamoto M, Serizawa T, Shuto T, Akabane A, Higuchi Y, Kawagishi J (2014). Stereotactic radiosurgery for patients with multiple brain metastases (JLGK0901): a multi-institutional prospective observational study. Lancet Oncol.

[8] Nicosia L, Figlia V, Napoli G, Giaj-Levra N, Ricchetti F, Rigo M, Lunardi G, Tomasini D, Bonu M, Corrdini S, Ruggieri R, Alongi F (2020). Repeated Stereotactic Radiosurgery (SRS) Using a Non-Coplanar Mono-Isocenter (HyperArc) Technique Versus Upfront Whole-Brain Radiotherapy (WBRT): A Matched-Pair Analysis. Clin Exp Metastasis.

[9] Tawbi HA, Forsyth PA, Algazi A, Hamid O, Hodi FS, Moschos SJ (2018). Combined nivolumab and ipilimumab in melanoma metastatic to the brain. N Engl J Med.

[10] Kluger HM, Chiang V, Mahajan A, Zito CR, Sznol M, Tran T (2019). Long-term survival of patients with melanoma with active brain metastases treated with pembrolizumab on a phase II trial. J Clin Oncol.

[11] Appelboom G, Detappe A, LoPresti M, et al. Stereotactic modulation of blood-brain barrier permeability to enhance drug delivery. Neuro Oncol. 2016;18(12):1601–9. 10.1093/neuonc/now137 PMC5744238 27407134

[12] Reits EA, Hodge JW, Herberts CA, Groothuis TA, Chakraborty M, Wansley EK, et al. Radiation modulates the peptide repertoire, enhances MHC class I expression, and induces successful antitumor immunotherapy. J Exp Med. 2006;. 10.1084/jem.20052494 PMC3212727 16636135

[13] Kiess AP, Wolchok JD, Barker CA, Postow MA, Tabar V, Huse JT, et al. Stereotactic radiosurgery for melanoma brain metastases in patients receiving ipilimumab: Safety profile and efficacy of combined treatment. Int J Radiat Oncol Biol Phys [Internet]. Elsevier Inc.; 2015;92:368–375. Available from: 10.1016/j.ijrobp.2015.01.004. 10.1016/j.ijrobp.2015.01.004 PMC4955924 25754629

[14] Cohen-Inbar O, Shih HH, Xu Z, Schlesinger D, Sheehan JP. The effect of timing of stereotactic radiosurgery treatment of melanoma brain metastases treated with ipilimumab. J Neurosurg. 2017;127(5):1007–14. 10.3171/2016.9.JNS161585 28059663

[15] Chen L, Douglass J, Kleinberg L, Ye X, Marciscano AE, Forde PM (2018). Concurrent immune checkpoint inhibitors and stereotactic radiosurgery for brain metastases in non-small cell lung Cancer, melanoma, and renal cell carcinoma. Int J Radiat Oncol biol Phys.

[16] Keir ME, Liang SC, Guleria I, Latchman YE, Qipo A, Albacker LA (2006). Tissue expression of PD-L1 mediates peripheral T cell tolerance. J Exp Med.

[17] Minniti G, Anzellini D, Reverberi C, Cappellini GCA, Marchetti L, Bianciardi F (2019). Stereotactic radiosurgery combined with nivolumab or Ipilimumab for patients with melanoma brain metastases: Evaluation of brain control and toxicity. J ImmunoTher Cancer.

[18] Helis CA, Hughes RT, Glenn CW, Lanier CM, Masters AH, Dohm A, et al. Predictors of Adverse Radiation Effect in Brain Metastasis Patients Treated with Stereotactic Radiosurgery and Immune Checkpoint Inhibitor Therapy. Int J Radiat Oncol Biol Phys [Internet]. Elsevier Inc.; 2020; Available from: 10.1016/j.ijrobp.2020.06.057. 10.1016/j.ijrobp.2020.06.057 32615262

[19] Martin AM, Cagney DN, Catalano PJ, Alexander BM, Redig AJ, Schoenfeld JD (2018). Immunotherapy and symptomatic radiation necrosis in patients with brain metastases treated with stereotactic radiation. JAMA Oncol.

[20] Pike LRG, Bang A, Ott P, Balboni T, Taylor A, Catalano P (2017). Radiation and PD-1 inhibition: Favorable outcomes after brain-directed radiation. Radiother Oncol.

[21] Kotecha R, Kim JM, Miller JA, et al. The impact of sequencing PD-1/PD-L1 inhibitors and stereotactic radiosurgery for patients with brain metastasis. Neuro Oncol. 2019;21(8):1060–8. . 10.1093/neuonc/noz046 PMC6682202 30796838

[22] Qian JM, Yu JB, Kluger HM, Chiang V (2016). 1 Title: Timing and type of immune checkpoint therapy affects early radiographic response of melanoma brain metastases to stereotactic radiosurgery Jack M. Qian, B.S. Cancer.

[23] Lehrer EJ, Peterson J, Brown PD, Sheehan JP, Quiñones-Hinojosa A, Zaorsky NG, et al. Treatment of brain metastases with stereotactic radiosurgery and immune checkpoint inhibitors: an international meta-analysis of individual patient data. Radiother Oncol, Available from. 2019;130:104–12 10.1016/j.radonc.2018.08.025. 10.1016/j.radonc.2018.08.025 30241791

[24] Eljalby M, Pannullo SC, Schwartz TH, Parashar B, Wernicke AG (2019). Literature review optimal timing and sequence of immunotherapy when combined with stereotactic radiosurgery in the treatment of brain metastases. World Neurosurg.

[25] Kiess AP, Wolchok JD, Barker CA, Postow MA, Tabar V, Huse JT (2015). Stereotactic radiosurgery for melanoma brain metastases in patients receiving ipilimumab: Safety profile and efficacy of combined treatment. Int J Radiat Oncol Biol Phys.

[26] Parvez K, Parvez A, Zadeh G (2014). The diagnosis and treatment of pseudoprogression, radiation necrosis and brain tumor recurrence. Int J Mol Sci.

